# Disparities in the gut metabolome of post-operative Hirschsprung's disease patients

**DOI:** 10.1038/s41598-021-95589-0

**Published:** 2021-08-09

**Authors:** Vera Plekhova, Ellen De Paepe, Katrien Van Renterghem, Myriam Van Winckel, Lieselot Y. Hemeryck, Lynn Vanhaecke

**Affiliations:** 1 Department of Veterinary Public Health and Food Safety, Faculty of Veterinary Medicine, Ghent University, Merelbeke, Belgium; 2 Department of Pediatric Surgery, Ghent University Hospital, Ghent University, Ghent, Belgium; 3 Department of Pediatrics and Medical Genetics, Ghent University Hospital, Ghent University, Ghent, Belgium; 4 School of Biological Sciences, Queen’s University Belfast, Belfast, UK

**Keywords:** Metabolomics, Enteric neuropathies

## Abstract

Hirschsprung's disease (HD) is a congenital structural abnormality of the colon seen in approximately 1 to 5000 live births. Despite surgical correction shortly after presentation, up to 60% of patients will express long-term gastrointestinal complaints, including potentially life-threatening Hirschsprung-associated enterocolitis (HAEC). In this study fecal samples from postoperative HD patients (n = 38) and their healthy siblings (n = 21) were analysed using high-resolution liquid chromatography—mass spectrometry aiming to further unravel the nature of the chronic gastrointestinal disturbances. Furthermore, within the patient group, we compared the faecal metabolome between patients with and without a history of HAEC as well as those diagnosed with short or long aganglionic segment. Targeted analysis identified several individual metabolites characteristic for all HD patients as well as those with a history of HAEC and long segment HD. Moreover, multivariate models based on untargeted data established statistically significant (p < 0.05) differences in comprehensive faecal metabolome in the patients’ cohort as a whole and in patients with a history of HAEC. Pathway analysis revealed the most impact on amino sugar, lysine, sialic acid, hyaluronan and heparan sulphate metabolism in HD, as well as impaired tyrosine metabolism in HAEC group. Those changes imply disruption of intestinal mucosal barrier due to glycosaminoglycan breakdown and dysbiosis as major metabolic changes in patients’ group and should be further explored for potential diagnostic or treatment targets.

## Introduction

Hirschsprung’s disease (HD, aganglionic megacolon) is a congenital anomaly with an incidence of 1 in 5,000 live births. The condition is caused by the absence of parasympathetic intrinsic ganglion cells in the submucosal and myenteric plexuses of the hindgut, due to the premature arrest of the craniocaudal migration of vagal neural crest cells between the 5th and 12th week of gestation^1,2^. Indeed, the condition can be limited to the distal part of the sigmoid colon (short segment HD) but also extend to the splenic flexure (long segment HD) or affect the entire colon in case of total colonic aganglionosis (TCA)^3^. In newborns, the disease presents itself as delayed passage of meconium, abdominal distention, vomiting and neonatal enterocolitis. Later in life, constipation, chronic abdominal distention, persistent vomiting and failure to thrive can become dominating symptoms^4,5^. Diagnosis of HD is confirmed by rectal biopsy and followed by surgical treatment using a “pull-through” procedure, where the aganglionic segment is resected and the healthy intestine reconnected to the anal margin. The aganglionic internal sphincter is preserved in order to enable anal continence^6^.

Despite definitive treatment, up to 80% of patients will report some form of bowel disfunction associated with HD complications sooner or later in life^7^. Frequently reported problems include constipation or fecal incontinence, bloating, diarrhea and recurrent Hirschsprung-associated enterocolitis (HAEC)^6,8^. Although all HD patients can develop disease-associated conditions, a number of factors, including long-segment disease, can increase that risk^9^. HAEC is a complication unique to HD patients, seen in 6 to 60% of children before and up to 37% after surgical intervention^8^. Substantial differences in reported cases of HAEC are mostly due to the absence of a unified diagnostic approach that can accurately and correctly recognize light and mild cases, both lacking pathognomonic signs. Absence of clear diagnostic criteria for HAEC causes undertreatment and further progression of the disease, contributing to decreased quality of life, high morbidity and increased medical costs^10^.

To date, several studies have focused on the long-term post-surgical complications of HD and factors that might explain HAEC etiology and incidents. Increasing evidence points towards microbiome alterations as a cause or contributing factor in the development of enterocolitis in HD patients^11,12^. Moreover, a distinctly different gut microbial pattern has been demonstrated in HAEC patients, even after symptom remission, suggesting that enterocolitis may either be caused by or result in a disruption of the patient’s uniquely adapted intestinal microbiome^13^. In addition to direct evidence of microbial shifts resulting from intestinal dysbiosis, Demehri et al.^14^ demonstrated suppressed microbial short-chain fatty acid (SCFA) formation in HD patients with a history of enterocolitis. SCFAs, mainly butyrate, propionate and acetate, are crucial for normal colonocyte function and a decrease in their concentration has been implicated in the development of inflammatory bowel disease (IBD)^15^. In addition, HD patients and in particular patients with a long aganglionic segment have been shown to have a higher risk of developing IBD later in life^16^. Several studies report decreased mucin production and turnover in HD patients as a potential predisposing factor for the development of HAEC^17,18^. Indeed, decreased production and turnover of mucins in the protective intestinal mucus layer leads to an impaired intestinal barrier function and contributes to intestinal inflammation by promoting closer contact between host cells and the intestinal microbiota^19^. Furthermore, several microbiome-derived inflammatory markers (including ASCA IgA and OmpC antibodies) were retrieved in serum of both Chrohn’s disease and HD patients with HAEC episodes^20^. The latter suggests similarities between the two pathologies at the level of chronic intestinal inflammation, provoked and maintained by intestinal dysbiosis, and also specifically points towards host-microbiome interaction playing an essential role in triggering and sustaining gut inflammation in HD patients susceptible to HAEC^20^. None of those markers, however, allow to fully explain or predict HAEC episodes and long-term surgery outcomes in HD patients, pointing towards interference from multiple and some yet unknown factors in the manifestation of this pathology^21^.

As a measure to account for the multifactorial nature of some complex gastrointestinal diseases, like e.g. colorectal cancer and IBD, a growing number of studies employ metabolomics for the investigation and comprehensive characterization of biochemical processes within a specific pathophysiological state^22,23^. Analysis of the fecal metabolome, representing the total sum of small molecules produced in biochemical reactions by the human host and gut microbiota under the influence of genetic, environmental and dietary factors, allows to outline and broadly characterize any gut-related pathophysiological condition. As such, metabolomics does not only enhance our knowledge on the etiology and pathogenesis, but also represents a powerful tool for biomarker discovery, bidding new opportunities for e.g. the development of diagnostic tests^24^. Additionally, non-invasive sampling of feces is more favorable to conventional invasive sampling of blood, particularly in young children^25,26^.

In this work, we present metabolomics data from stool samples obtained from post-operative HD patients and their healthy siblings. Additionally, within the patients’ group we investigated metabolic shifts in children with and without a history of HAEC as well as those diagnosed with short or long aganglionic segment in search for disease-specific biochemical alterations that can aid to unravel the pathophysiology of HAEC and identify novel diagnostic or prognostic biomarkers.

## Results

Comprehensive metabolomics analysis of fecal extracts produced 27,906 positively and 14,854 negatively charged metabolite features reflecting the individuals’ unique fingerprints. Accurate evaluation of specified targeted components (291 standards) allowed the identification of several metabolites responsible for biochemical differences within the investigated groups. Targeted and untargeted data were integrated to elucidate the main pathways underlying the metabolic differences in the investigated groups.

### Metabolic differences between post-operative HD patients and their healthy siblings

#### Targeted metabolite profiling

**Figure 1:**
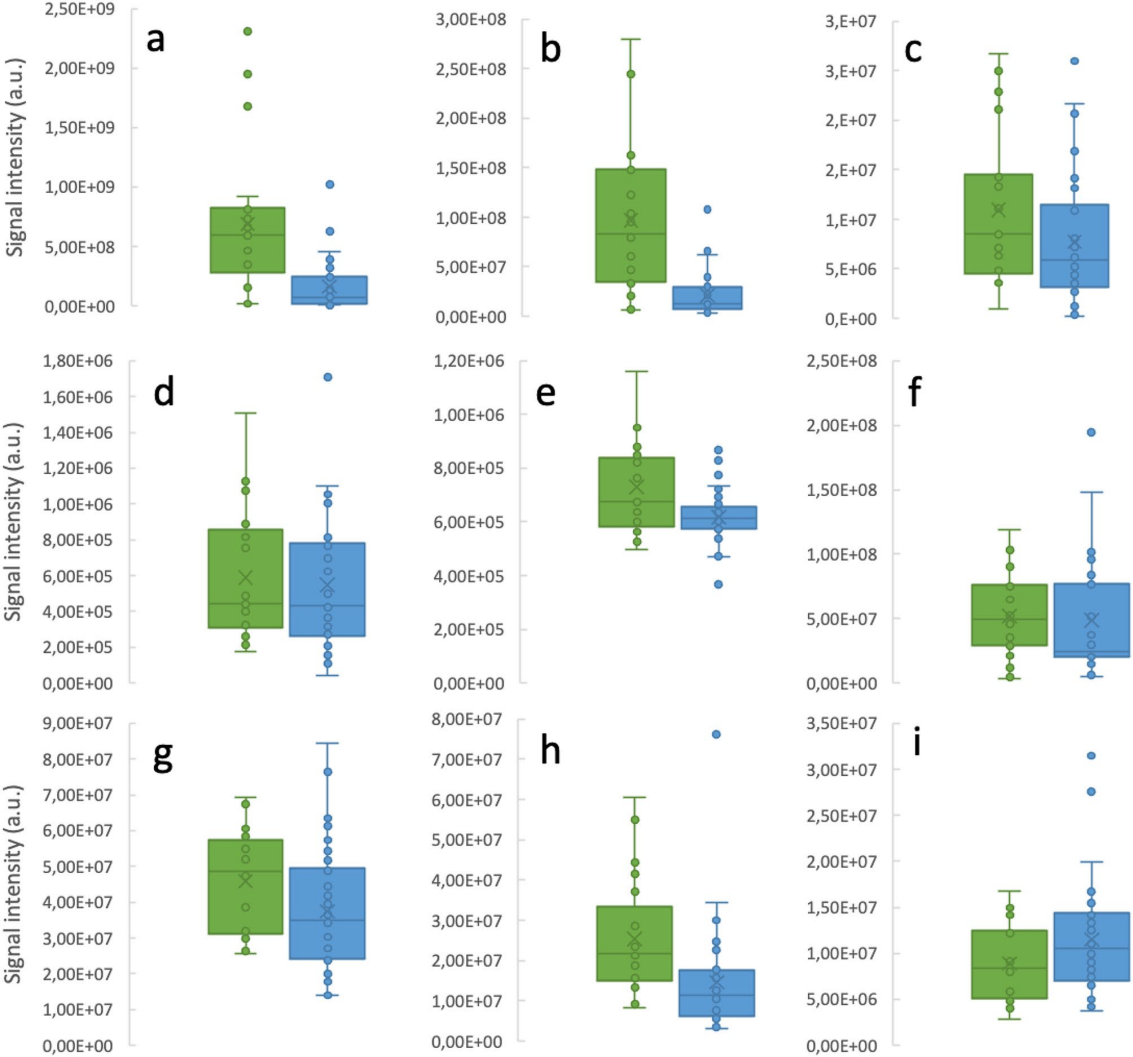
Boxplots representing signal intensities of significantly (p < 0.05) altered metabolites in HD patients (green, n = 34) *vs.* healthy controls (blue, n = 21) for pipecolinic acid (**a**), N-acetylneuraminic acid (**b**), N-acetylglucosamine (**c**), glutamic acid (**d**), isovaleric acid (**e**), octan-3-one (**f**), fructose (**g**), serine (**h**) and dopamine (**i**).

Of the 142 metabolites retrieved and identified, 9 were significantly different between the HD patient group and their healthy siblings (Fig. 1). Decreased concentrations were observed for pipecolinic acid (p < 0.001), *N*-acetylneuraminic acid (p = 0.005), *N*-acetylglucosamine (p = 0.017), glutamic acid (p = 0.030), isovaleric acid (p = 0.024), octan-3-one (p = 0.023), fructose (p = 0.027) and serine (p = 0.043). Dopamine concentrations (p = 0.034) were increased in HD patients as opposed to control group.

#### Untargeted metabolic fingerprinting

**Figure 2:**
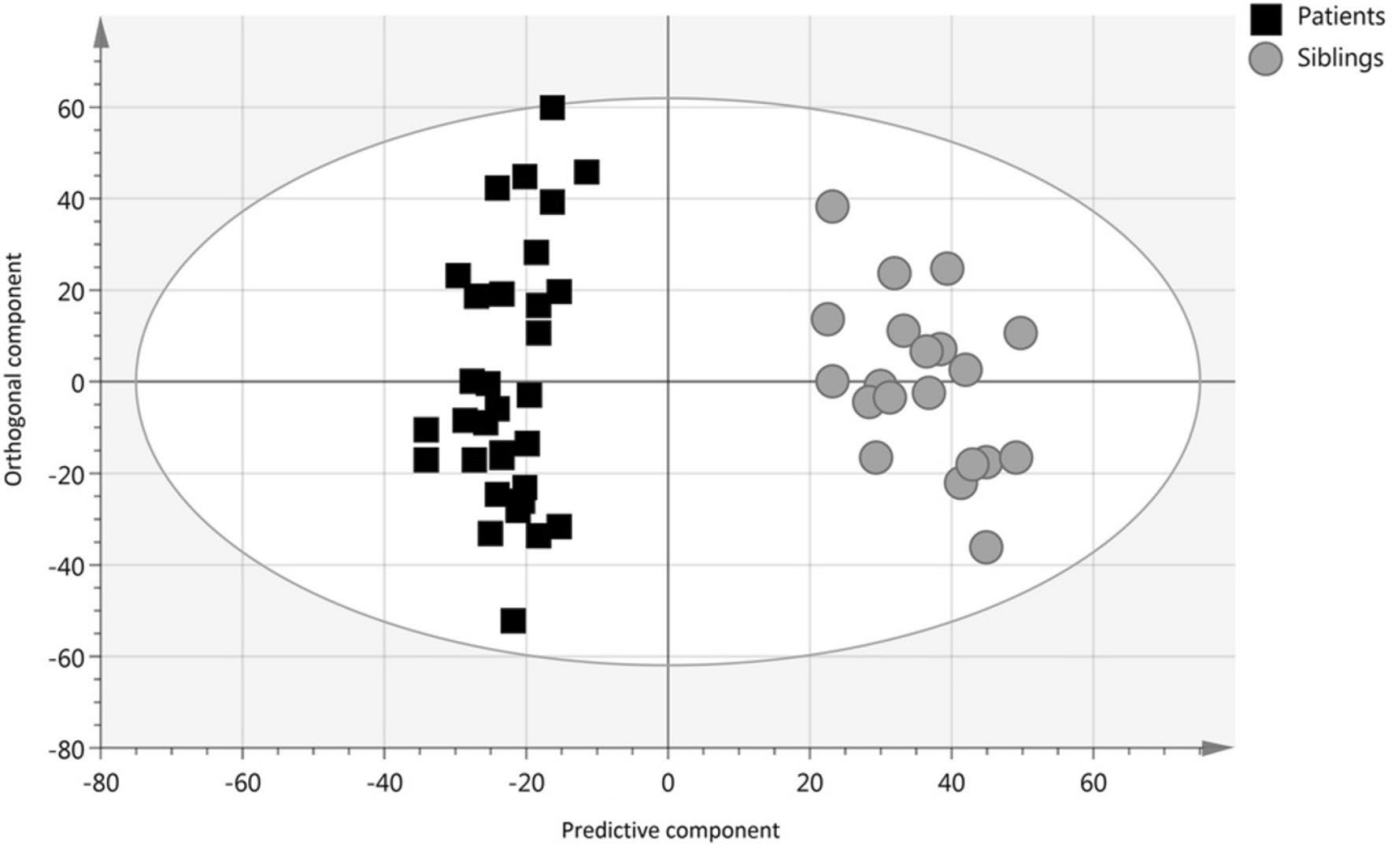
Fecal metabolome OPLS-DA scatter plot of HD patients (n = 34) *vs.* healthy siblings (n = 21). The validity of the OPLS-DA model was confirmed (R^2^Y(cum) = 0.784, Q^2^(cum) = 0.505, CV-ANOVA p-value = 2.83e^−06^ and good permutation testing).

The principal component analysis (PCA) revealed the presence of four outliers identified by 95% Hotelling's T2 criterion and residual distance of the observation to its projection. All four outliers belonged to patients with total colonic aganglionosis and were excluded from further evaluation in this multivariate model. Subsequently, a valid OPLS-DA regression model was constructed, enabling supervised group separation (Fig. 2)^27^. Among features contributing the most to group separation, it was possible to list 10 features of interest (Supplementary Table S2). Two of these features were downregulated in HD patients and could be tentatively identified as R-lipoic acid and N2-oxalylarginine.

In addition, the same approach was applied to test whether or not dietary habits affected the fecal metabolome of post-operative HD patients *vs*. their healthy siblings. As no identical features were detected among the ones contributing most to group separation between post-operative HD patients and their healthy siblings (VIP > 1.5), it was concluded that differences in the metabolic fingerprint due to diet did not interfere with disease classification.

#### Pathway analysis

**Figure 3:**
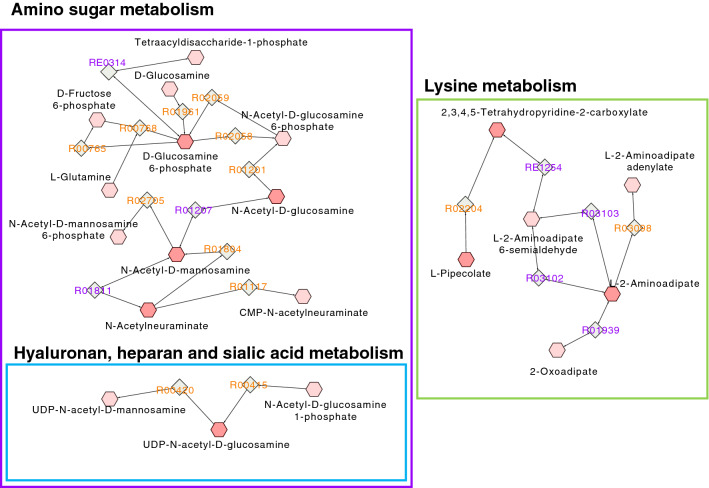
Overview of metabolic pathways underlying the differentiation of the fecal metabolome of HD patients (n = 34) and their healthy siblings (n = 21). The network demonstrates connected metabolic features (hexagons) with corresponding enzyme reaction (diamonds) within statistically significant (p < 0.05) pathways.

Pathway analysis was based on 1870 unique metabolites of interest (CV-ANOVA p-value < 0.05). Subsequent pathway visualization (Fig. 3) included both empirically identified untargeted features (Supplementary materials Table S3) as well as significant metabolites from targeted analysis. With adjustment for multiple comparisons, the main differences between the patient’s and sibling’s fecal metabolic fingerprints could be attributed to several catabolic pathways. The most affected was the lysine pathway (p = 0.024). Amino sugar metabolism was significantly (p = 0.047) downregulated in HD patients, as supported by the targeted data where lower glutamic acid, N-acetylglucosamine, N-acetylneuraminic acid and fructose concentrations were measured in the patient group. Also, in HD patients, pathways associated with glycosaminoglycan (GAG) degradation, including hyaluronan (p. = 0.049) and heparan sulfate degradation pathway (p = 0.049) were upregulated.

### Metabolic differences between HD patients with and without a history of HAEC

#### Targeted metabolite profiling

**Figure 4:**
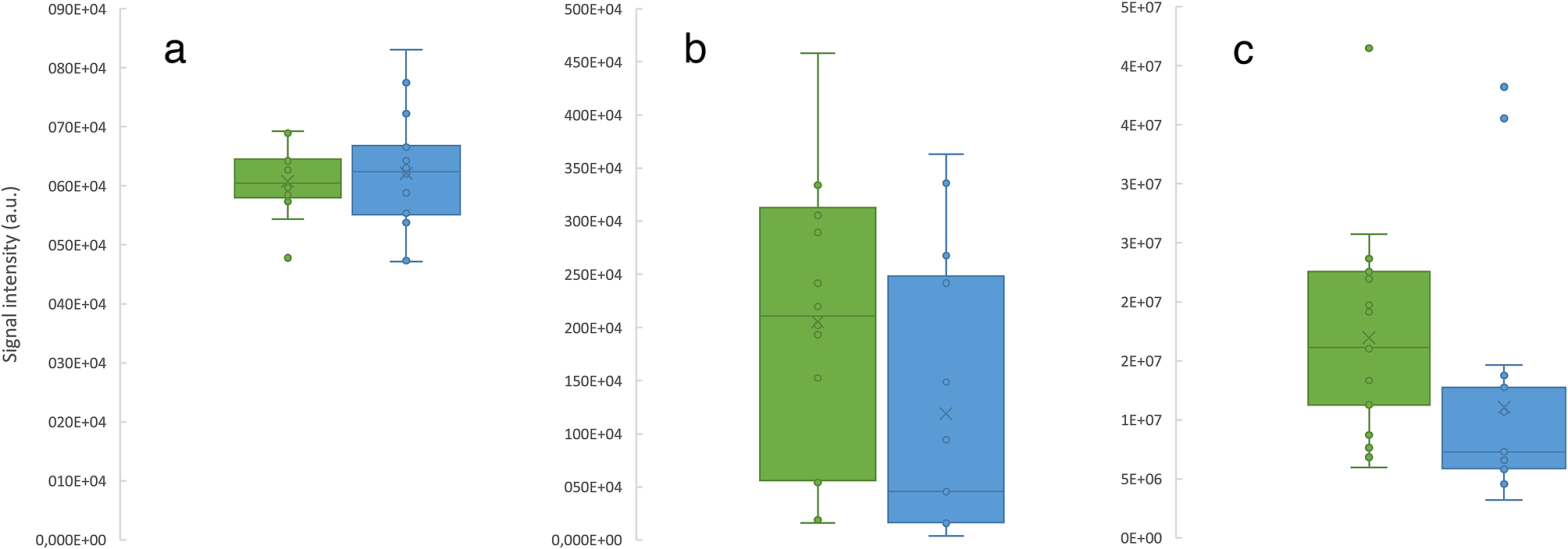
Boxplots representing signal intensities of significantly (p < 0.05) altered metabolites in patient groups with (green, n = 19) and without (blue, n = 18) history of Hirschsprung-associated enterocolitis for ethyl pentyl ketone (**a**), 4-methyl-3-penten-2-one (**b**) and trans-4-hydroxy-l-proline (**c**).

Within the HD patient cohort, patients who previously endured HAEC episodes showed increased levels of trans-4-hydroxy-l-proline (p = 0.010) and 4-methyl-3-penten-2-one (p = 0.049) as well as a decrease in ethyl pentyl ketone (p = 0.046) compared to patients without a history of HAEC (Fig. 4).

#### Untargeted metabolic fingerprinting

**Figure 5:**
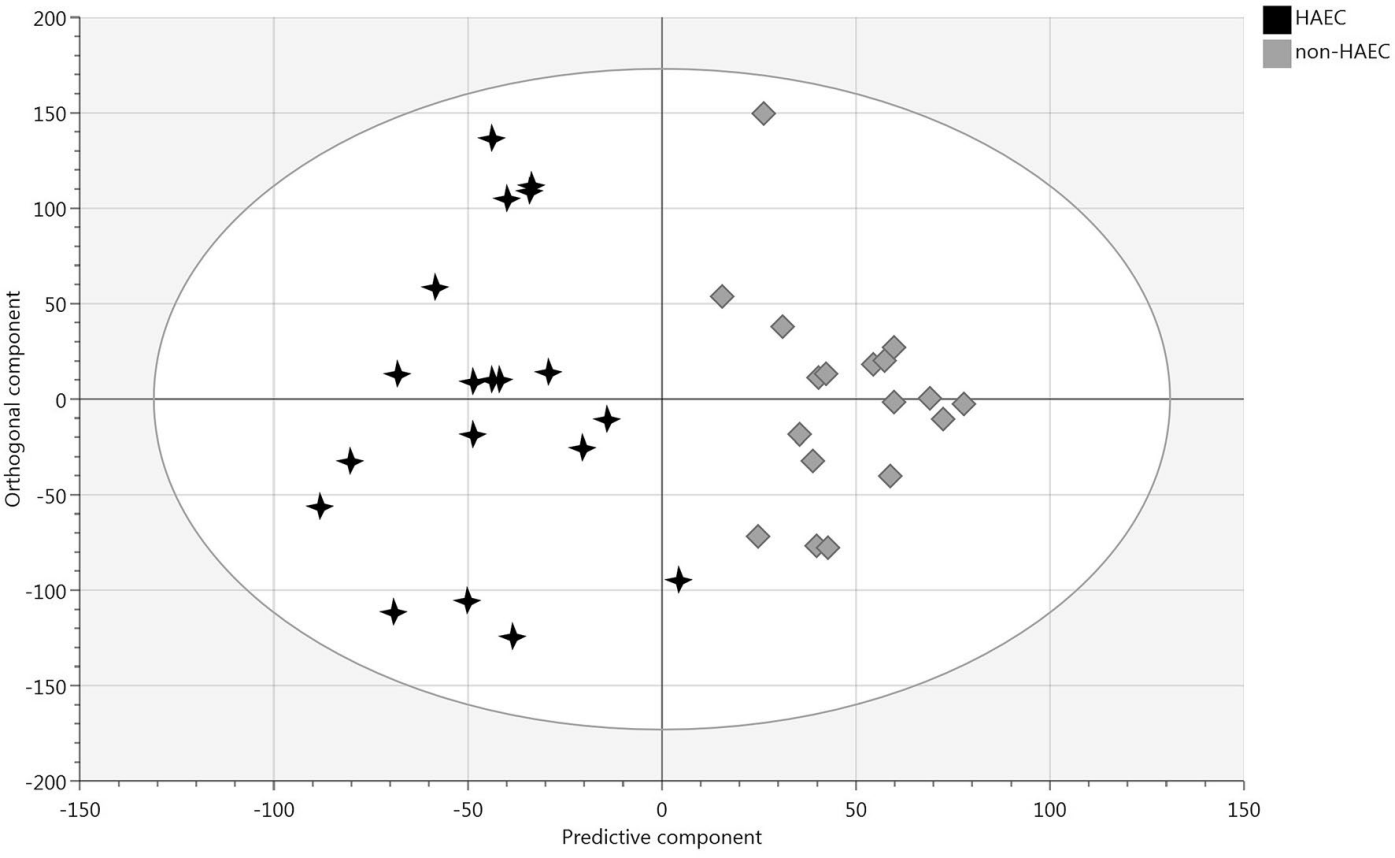
OPLS-DA scatter plot showing separation between patients with (n = 19) and without history of HAEC (n = 18). The validity of the model was confirmed (R^2^Y(cum) = 0.844, Q^2^(cum) = 0.55, CV-ANOVA p-value = 2.73e^−05^ and good permutation testing).

Good descriptive and predictive properties of the OPLS-DA model, i.e., R^2^Y of 0.844, Q^2^ of 0.55, CV-ANOVA p-value of 2.73e^−05^ and acceptable permutation test, demonstrates a significant difference in the comprehensive fecal metabolic fingerprint of patients with and without a history of HAEC (Fig. 5). In total, 19 discriminating features of interest could be listed for the enterocolitis group (Supplementary Table S4), revealing several dipeptides consisting of phenylalanine as common amino acid: i.e. glutaminylphenylalanine, phenylalanylvaline; as well as 6-hydroxymelatonin and 1-methylnicotinamide.

#### Pathway analysis

Pathway analysis was based on 1782 features with a CV-ANOVA p-value < 0.05. Among the affected pathways, a significant difference between patients based on their HAEC history was revealed for tyrosine metabolism (p = 0.0028).

### Metabolic differences between HD patients with short and long aganglionic segment

#### Targeted metabolite profiling

**Figure 6:**
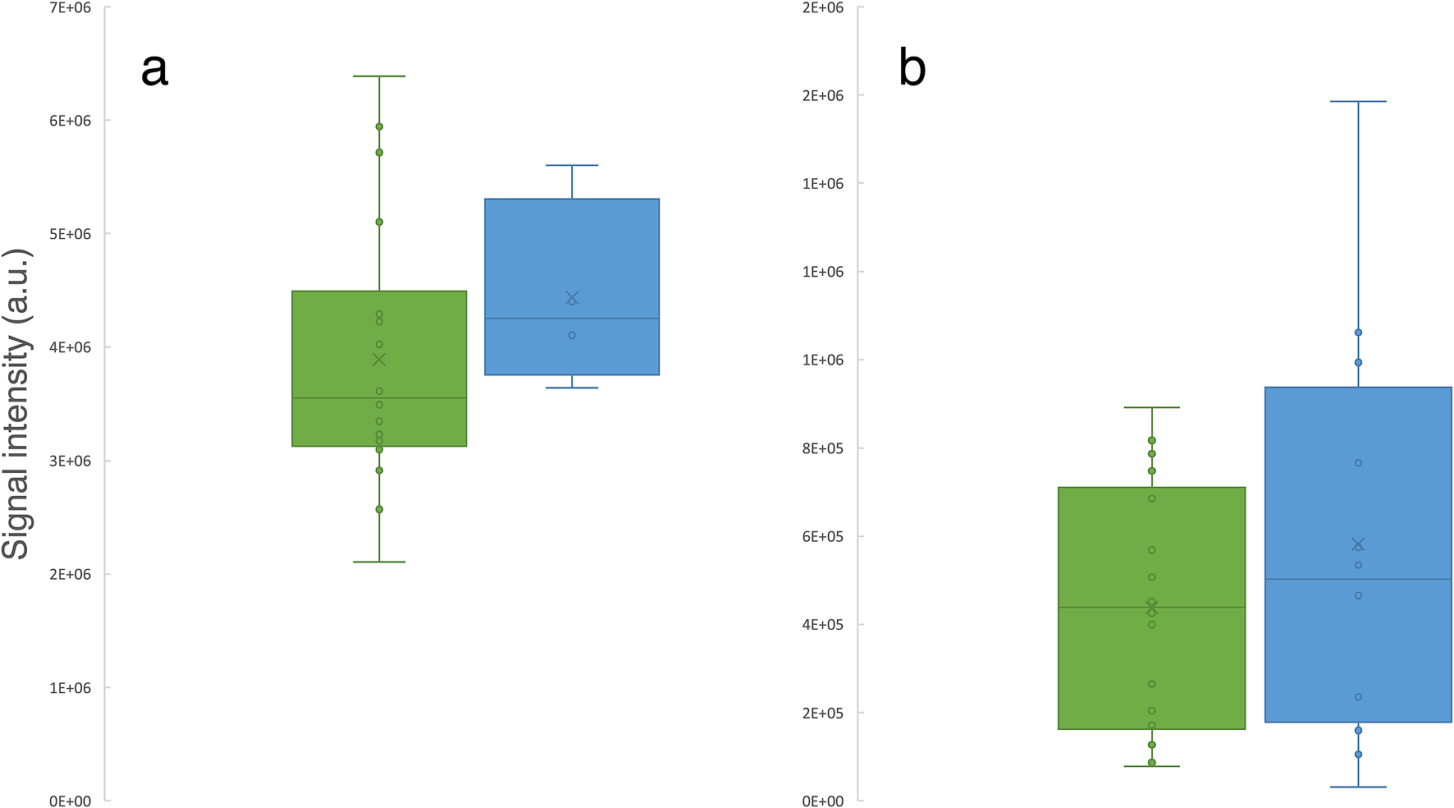
Boxplots representing signal intensities of significantly (p < 0.05) altered metabolites in patient groups diagnosed with short aganglionic segment (green, n = 24) and with either long aganglionic segments or total colonic aganglionosis (blue, n = 14) for 3-methyl-2cyclohexene-1-one (**a**) and 2-methyl-3-pentanon (**b**).

In patients with long segment HD (including patients with TCA), 3-methyl-2-cyclohexen-1-one (p = 0.025) and 2-methyl-3-pentanone (p = 0.043) were significantly upregulated in contrast to patients diagnosed with short aganglionic segment (Fig. 6).

#### Untargeted metabolic fingerprinting

OPLS-DA models based on patient’s aganglionic segment length showed only borderline significant validation parameter values with average prediction abilities^28^. The following parameters were obtained from the OPLS-DA model comparing short segment (n = 24) *vs.* long segment HD including total colonic aganglionosis (n = 14): R^2^Y(cum) = 0.997, Q^2^(cum) = 0.452, CV-ANOVA p-value = 0.014 and acceptable permutation test. However, when evaluating the PCA-X model for natural clustering and outlier detection as described under 2.1.2, it was found that the patients with total colonic aganglionosis (TCA) differed significantly from the other patients and the healthy cohort. Namely, three out of four TCA patients acted as outliers based on Hotteling's 95% T2 confidence limit and 4 out of 4 observations were classified as outliers based on residual distance of the observation to its projection (DMod)^29,30^. Both the PCA-X score and Distance to model plots can be consulted in the Supplementary material (Fig. S1).

Further, an OPLS-DA model excluding TCA patients was constructed to evaluate differences with short (n = 24) and long (n = 10) segment aganglionosis. Despite exclusion of potential outliers, the performance of the model did not significantly improve, with R^2^Y(cum) = 0.849, Q^2^(cum) = 0.326, CV-ANOVA p-value = 0.026 and acceptable permutation test. As no fully validated untargeted models were constructed, no discriminating feature extraction was performed for pathway analysis.

## Discussion

In this study, several gut microbiome derived metabolites and fermentation pathways, as a direct reflection of gut microbiome functionality, were established to be discriminating for the fecal metabolome of HD patients compared to their healthy siblings. Indeed, involvement of the gut microbiome in the pathogenesis of post-operative HD complications is in line with previously published reports^11,12^. This can be viewed as a consequence of the (partial) colon resection, as the latter drastically changes the intestinal microenvironment required for maintaining a healthy microbiome. Lysine catabolism was upregulated in HD patients and has previously been associated with inadequate bacterial butyrate production in the large bowel, in which lysine represents alternative to carbohydrates as the main substrates for SCFA production^31,32^. Since a drop in amino sugars (cfr. the reported decrease in N-acetylglucosamine and N-acetylneuraminic acid) has also been linked to impaired carbohydrate digestion resulting from dysbiosis^33^, these findings points towards microbiome-related disruptions in HD patients. This is in line with previous reports on the distinctive microbiome^12^ and SCFA profile in HD patients^14^ as a potential contributing factor in the development of enterocolitis^11,12^. A distinctly different gut microbial pattern has e.g. indeed been demonstrated in HAEC patients, even after symptom remission, suggesting that enterocolitis may either be caused by or result in a disruption of the patient’s uniquely adapted intestinal microbiome^13^.

Equally important is the involvement of dysbiosis in altering the intestinal mucus layer. Several detected alterations point towards an increased degradation of major mucus components such as GAGs, regardless of patient’s HAEC history. This mucus depletion has been investigated in light of several other gastro-intestinal diseases, such as ulcerative colitis, and is known to be a factor supporting and promoting gut inflammation^34^. Moreover, several previous studies have reported decreased mucin production and turnover specifically in HD patients with a possible link to developing enterocolitis^17,18^. Here we find upregulated hyaluronan and heparan sulfate degradation in HD patients, involved in GAG turnover. GAGs together with mucopolysaccharides are essential components of the protective intestinal mucus layer^35,36^. In the healthy colon, simple sugars derived from GAGs provide binding sites and nutrition for commensal microbiota^37^. However, in case of mucus barrier dysfunction or damage, the supplementary nutrient source provided by degradation of GAGs will contribute to bacterial overgrowth^35^. In addition, the degradation of GAGs is accompanied by the release of small hyaluronan fragments, known to stimulate leukocyte adhesion and inflammatory cytokine production. When overdriven it has been associated with chronic gut inflammation^38^. Additional evidence supporting the theory of increased microbial mucin degradation in HD patients could be found in significantly altered sialic or N-acetylneuraminic acid metabolism^39^, since many commensal bacteria produce sialidases to harvest sialic acid at the terminal regions of mucin molecules. Mucins, as well as GAGs, are essential for a normal activity of the local immune system and epithelial barrier integrity^40^. Additionally, the mucus layer provides a natural habitat for the residing gut microbiota and has been suggested to impact on colon-specific microbiome formation^41^. Loss of the mucus barrier stimulates passive intestinal absorption and allows closer contact between host immune cells and bacterial antigens. An increased rate of mucin degradation and a decrease in epithelial barrier function are therefore additional predisposing factors for maintaining an intestinal inflammatory state^36,42^. In other words, metabolic and enzymatic shifts introduced by a dysregulated gut microbiome induce increased mucin deterioration, which in turn creates favorable circumstances for further development of dysbiosis. In HD patients, colonic dysbiosis has been appointed as one of the major drivers of the development of enterocolitis^43,44^. However, alterations in microbial composition can also be responsible for more chronic HD complications, such as constipation and abdominal distention^45^. Future research is warranted to confirm that the observed metabolic alterations are coinciding the hypothesized shifts in microbiome composition and would thus benefit from an integrative omics approach.

Among evidence of dysbiosis and alterations in mucus turnover in HD patients in this study, HAEC patients presented additional metabolic changes including increased tyrosine catabolism, a decreased Trans-4-hydroxy-l-proline (Hyp) degradation and alterations in specific volatile compounds (ethyl pentyl ketone and 4-methyl-3-penten-2-one). Hyp is the common product of anaerobic proline transformation utilized by the gut microbiota, primarily by *Clostridium* spp.^46^. Clostridia are widely abundant commensal species that have been shown to populate specific regions of the gastrointestinal tract, mainly the ascending colon^47^, where they act as major butyrate-producing species^48^. Increased Hyp concentrations may imply a decrease in Hyp-utilizing microbiota and can explain lower SCFAs concentrations in HAEC patients, as previously demonstrated^14^. Further, 4-methyl-3-penten-2-one—increased in patients with a history of HAEC—and ethyl pentyl ketone—decreased in patients with a history of HAEC—are volatile compounds that can originate from the microbiome, but also from nutrition (host metabolite). In pediatric celiac disease patients, 4-methyl-3-penten-2-one and ethyl pentyl ketone were shown to be augmented in urine and saliva, and associated with shifts in the gut microbiome^49,50^. Interestingly, similarities of HAEC biomarkers with pediatric celiac disease markers hint towards similarities in pathophysiology and warrant further investigation into potential diagnostic and/or management correlations, furthermore, providing promising predictive, diagnostic and/or prognostic marker candidates.

Among the affected pathways, a significant difference between patients based on their HAEC history was only revealed for tyrosine metabolism. Aromatic amino acids such as tyrosine have been shown to act as signaling molecules within the microbiome-host cross-talk^51^. Moreover, phosphorylation of tyrosine by tyrosine kinases is involved in multiple aspects of cell metabolism regulation. Tyrosine kinase inhibitors have demonstrated potential to subdue intestinal inflammation by reducing cytokine expression in response to bacterial stimulation and represent promising therapeutical targets for colitis patients^52^. Although the same process of gut-microbiome tyrosine dependent interaction can be suggested for HAEC patients, the exact mechanism and consequences are yet to be determined.

In patients with long segment HD (including patients with TCA), 3-methyl-2-cyclohexen-1-one (p = 0.025) and 2-methyl-3-pentanon (p = 0.043) were found to be upregulated. Both metabolites represent volatile organic compounds commonly detected in fecal samples. In literature, an increase in 2-methyl-3-pentanone has been reported in patients with *Campylobacter jejuni* infections, while 3-methyl-2-cyclohexen-1-one has been observed in patients with *Clostridium difficile* infections^53^. Although more data are needed to determine the significance of this increase in long segment HD patients, it is noteworthy that microbial infections or dysbiosis can be again assigned as probable cause. Indeed, a significant disparity in the comprehensive metabolic fingerprint of long and short segment HD was observed, as unsupervised data exploration based on PCA-X clearly separated TCA patients as outlying observations in the entire cohort. Those findings imply that the fecal metabolome of TCA patients differs markedly not only from healthy subjects, but also from patients diagnosed with short and long aganglionic segments. Although we are unable to draw conclusions about the nature of those changes due to the small sample size of the TCA group (n = 4), these preliminary data can act as proof-of-concept for further research focusing on TCA patients.

## Material and methods

### Subjects and samples

#### Participants

Participants included 38 post-surgery HD patients and 21 healthy siblings recruited at the Ghent University Hospital (Prof. Van Winckel and Dr. Van Renterghem). Enrolled patients were diagnosed with a short aganglionic segment (n = 24), long segment (n = 10), and total colonic aganglionosis (n = 4) that underwent the same surgical treatment (colonic pull through procedure). Fecal samples were collected at the earliest one year after surgery to ensure adequate recovery. Within the HD cohort, 19 patients (50%) reported to be diagnosed with at least one episode of HAEC over the course of the disease. A more detailed overview of the investigated cohort is included in supplementary material Table S1. This study was approved by the Ghent University Hospital ethical committee (EC 2015/1193) and executed in accordance with relevant guidelines and regulations. The parental informed consent (EC 2015/1426) was obtained from each participant in accordance with the Declaration of Helsinki.

#### Sample handling

Fecal samples were obtained through spontaneous defecation or routine enema in those unable to spontaneously defecate and stored at − 20 °C until pick-up (up to maximum two weeks). Following cooled transport (dry ice) to the lab, samples were briefly stored at − 80 °C, thereafter lyophilized and homogenized in preparation of a more long-term storage at − 80 °C awaiting analysis.

### Metabolomics

#### Standards and reagents

Solvents for extraction and chromatographic separation were of LC–MS purity grade and purchased from VWR International (Merck) and Thermo Fisher Scientific. Ultrapure water was produced in-house using a Sartorius arium® 611 Ultrapure Type 1 Water System (Thermo Fisher Scientific). Chemical standards (a total of 291 metabolites and D-valine-d_8_ as internal standard) were obtained from diverse suppliers as reported previously^54^.

#### Sample analysis and quality assurance

Polar metabolome extraction, chromatographic separation and mass spectrometric analysis followed protocols described previously^26,55^.

Chromatographic separation was performed using an Accela UHPLC installation (Thermo Fisher Scientific) equipped with an Acquity HSS-T3 C18 column (1.8 μm, 150 mm × 2.1 mm, Waters) and VanGuard precolumn (1.8 μm, 5 mm × 2.1 mm, Waters) as reported previously^25,55^. Mass spectrometric detection was carried out on a hybrid quadrupole-Orbitrap high-resolution mass spectrometer (Q-Exactive™, Thermo Fisher Scientific) equipped with a heated electrospray ionization source (HESI-II)^26^.

In light of targeted analysis, a mixture of 291 analytical standards was prepared in a pooled matrix extract and analyzed alongside the samples, under the same instrumental conditions. Signal linearity was ensured by dilution series. To assure instrumental precision (mass deviation < 5 ppm), the instrument was calibrated in both polarity modes according to manufacturer recommendations. Pooled quality control (QC) samples consisting of equal aliquots of all sample extracts were analyzed alongside test specimens at equal intervals to monitor stability of instrument performance^56^. For the same purpose, the internal standard D-valine-d_8_ was added to all extracted samples at a concentration of 100 ng mL^−1^.

### Data analysis

#### Analysis of targeted metabolites

Targeted data processing, including peak detection and comparison with the reference standard solution mix, was performed using Xcalibur™ 3.0 (Thermo Fisher Scientific). Compound intensities were normalized to the intensity of the reference internal standard (D-valine-d_8_) in each sample. Univariate statistical analysis of targeted data was carried out with SPSS Statistics 26.0 (IBM). More specifically, one-way ANCOVA and Quade test with Bonferroni correction at a 5% significance level were applied for parametric and non-parametric analysis respectively. Results were adjusted for gender and age as covariates. Normality of distribution and equality of variances were assessed with Kolmogorov–Smirnov and Levine’s tests for each variable.

#### Analysis of untargeted metabolites

Sieve™ 2.2. software (Thermo Fisher Scientific) was used for untargeted data preprocessing. As primary parameters, a maximum retention time shift of 0.5 min, peak intensity threshold of 500.000 a.u., background signal-to-noise ratio of 10 and an *m*/z step size of 6 ppm were set. Multivariate statistical analysis was performed with SIMCA 15.0 (Sartorius, Germany). Data pre-treatment included log10 transformation and pareto-scaling to achieve approximate normal distribution^57^. Further, principal component analysis (PCA) was used for exploration of inherent sample clustering and outlier detection. Subsequently, orthogonal partial least squares discriminant analysis (OPLS-DA) was employed for supervised group separation. Validation of OPLS-DA models was based on the total variation explained by the model (R^2^Y) close to 1, model predictive properties (Q^2^) above 0.5, CV-ANOVA p-value < 0.05 and valid permutation testing (n = 100)^58^. For valid models, features contributing the most to group separation were selected based on a variable importance in projection (VIP) score > 1.5, a correlation |pcorr|> 0.5 and covariance |p|> 0.02 derived from the S-plot and Jack-knifed confidence intervals not including zero. Tentative identification was performed by HMDB database search, based on accurate *m/z* with a mass deviation tolerance of 5 ppm. Putative identities were cross-checked with the features’ ^12^C/^13^C isotope ratio, corresponding to Tier level 3 confidence level of identification^59,60^.

#### Combined pathway analysis

Pathway analysis was performed on the MetaboAnalyst 4.0 platform applying the “MS peaks to Pathways” tool based on the mummichog algorithm. Mass deviation tolerance was set at 5 ppm. Significance of metabolite identification was determined as a p-value of permutation test between any other metabolites in the reference database. Pathway significance was determined by enrichment analysis with Holm adjustment for multiple comparisons^61^. Pathway visualization was performed on the MetScape 3 platform, based on the combined targeted and untargeted data.

## Supplementary Information

Supplementary Information.

## Data Availability

The experimental output and anonymized patient data will be made freely available upon publication.
