# Supplementary material for: Disparities in the gut metabolome of post-operative Hirschsprung's disease patients
Source: Sci Rep. 2021 Aug 9;11:16167. doi: 10.1038/s41598-021-95589-0 (PMC8352975; doi:10.1038/s41598-021-95589-0)
Supplement: Supplementary file 1 — Supplementary Information. [file 41598_2021_95589_MOESM1_ESM.pdf]

Disparities in the gut metabolome of post-operative Hirschsprung's disease patients

Vera Plekhova[1], Ellen De Paepe[1], Katrien Van Renterghem[2], Myriam Van Winckel[3], Lieselot Y. Hemeryck[1,a] and Lynn Vanhaecke[1,4,a]*.

Affiliations:

[1]Department of Veterinary Public Health and Food Safety, Faculty of Veterinary Medicine, Ghent University, Merelbeke, Belgium

[2]Department of Pediatric Surgery, Ghent University Hospital, Ghent University, Ghent, Belgium

[3]Department of Pediatrics and Medical Genetics, Ghent University Hospital, Ghent University, Ghent, Belgium

[4]Queen's University Belfast, School of Biological Sciences, Belfast, United Kingdom

[a]shared senior author

* corresponding author; lynn.vanhaecke@ugent.be

Table 1. Characteristics of participants cohort.

	Patients	Siblings
Number of participants	38	21
Median age (IQR), y.o.	6.5 (4.25)	6.5 (4.5)
Sex (male/female)	26/12	12/9
Type of aganglionic segment:		
Short segment	24	-
Long segment	10	-
Total colon aganglionosis	4	-
Time after surgery:		
- one to two years	3	-
- two to three years	7	-
- three years and longer	30	-

Table 2. Discriminating metabolic features of HD patients *vs*. healthy siblings as determined by OPLS-DA of the untargeted dataset.

m/z	RT (min.)	Polarity	VIP value	Adduct	Mass deviation (ppm)	Annotation
**189.0410**	6.07	+	2.36	M+H-H2O	1	(R)-lipoic acid
**256.9236**	5.39	-	2.17			ND
**274.0876**	6.17	+	2.13			ND
**286.1029**	7.91	-	2.08			ND
**274.1841**	4.34	+	1.96			ND
**388.2788**	11.2	-	1.94			ND
**318.0032**	3.31	+	1.94			ND
**204.9921**	5.39	+	1.91			ND
**493.1998**	1.31	+	1.87	2M+H	1	N2-Oxalylarginine
**370.0722**	8.63	-	1.85			ND
**391.1207**	4.93	-	1.61			ND

*ND = not determined

Table 3. Discriminating metabolic features for Hirschsprung's patients with a history of HAEC as opposed to those without, as determined by OPLS-DA of the untargeted dataset.

m/z	RT (min.)	Polarity	VIP value	Adduct	Mass deviation (ppm)	Annotation
147.5760	1.59	+	2.31	M+2H	0	Glutaminylphenylalanine
162.0947	8.33	+	2.22	M+H	1	Trimethylsilyl l-Alanine
129.0786	1.98	+	2.17	-	-	ND
154.6021	2.25	+	2.12	-	-	ND
119.0655	2.03	+	2.10	M+2H	1	Phenylalanylalanine
197.0613	1.68	+	2.08	-	-	ND
139.5968	4.27	+	2.06	M+2H	0	N-Acetylprocainamide
172.6021	5.28	+	2.04			ND
133.081	2.87	+	2.02	M+2H	0	Phenylalanylvaline
154.6021	2.25	+	2·02			ND
169.0324	3.18	+	1.99	M+2H	4	4-Hydroxy-5-(dihydroxyphenyl)-valeric acid-O-methyl-O-sulphate
125.0655	1.59	+	1.96	M+2H	1	6-Hydroxymelatonin
148.0777	1.59	+	1.96	-	-	ND
124.5735	1.58	+	1.93	-	-	ND
113.043	1.57	+	1.93	-	-	ND
173.1649	6.55	+	1.84	-	-	ND
168.0484	5.35	+	1.82	-	-	ND
133.5787	1.58	+	1.76	-	-	ND
178.0981	4.54	+	1.75	M+ACN+H	0	1-Methylnicotinamide

*ND = not determined

Table 4. Empirical metabolites showing network enrichment, as validated by multiple permutations extracted from the mummichog algorithm within significantly ($p < 0.05$) altered pathways.

Pathway	Emperical compound	KEGG code
Lysine metabolism	L-Glutamate	C00025
(p = 0.024)	D-Glutamic acid	C00217
	Glutamic acid	C00302
	L-4-Hydroxyglutamate semialdehyde	C05938
	(S)-2,3,4,5-Tetrahydropyridine-2-carboxylate	C00450
	Delta1-Piperideine-2-carboxylate	C04092
	N6-dimethyl-L-lysine	C05545
	4-Acetamidobutanoate	C02946
	6-Amino-2-oxohexanoate	C03239
	L-2-Aminoadipate	C00956
	Pipecolic acid	C00408
	4-Acetamidobutanal	C05936
Amino sugar metabolism	L-Glutamate	C00025
(p = 0.047)	D-Glutamate	C00217
	Glutamic acid	C00302
	L-4-Hydroxyglutamate semialdehyde	C05938
	N-Acetyl-D-glucosamine	C00140
	N-Acetyl-D-mannosamine	C00645
	N-Acetylgalactosamine	C01074
	N-Acetyl-D-galactosamine	C01132
	D-Glucosamine phosphate	C00352
	D-Galactosamine 6-phosphate	C06377
	Aminofructose 6-phosphate	C12214
Hyaluronan metabolism	N-Acetyl-D-glucosamine	C00140
(p = 0.049)	N-Acetyl-D-mannosamine	C00645
	N-Acetylgalactosamine	C01074
	N-Acetyl-D-galactosamine	C01132
Sialic acid metabolism	N-Acetyl-D-glucosamine	C00140
(p = 0.049)	N-Acetyl-D-mannosamine	C00645
	N-Acetylgalactosamine	C01074
	N-Acetyl-D-galactosamine	C01132
	N-Acetyl-D-glucosamine	C00140
Heparan sulfate degradation	N-Acetyl-D-mannosamine	C00645
(p = 0.049)	N-Acetylgalactosamine	C01074
	N-Acetyl-D-galactosamine	C01132

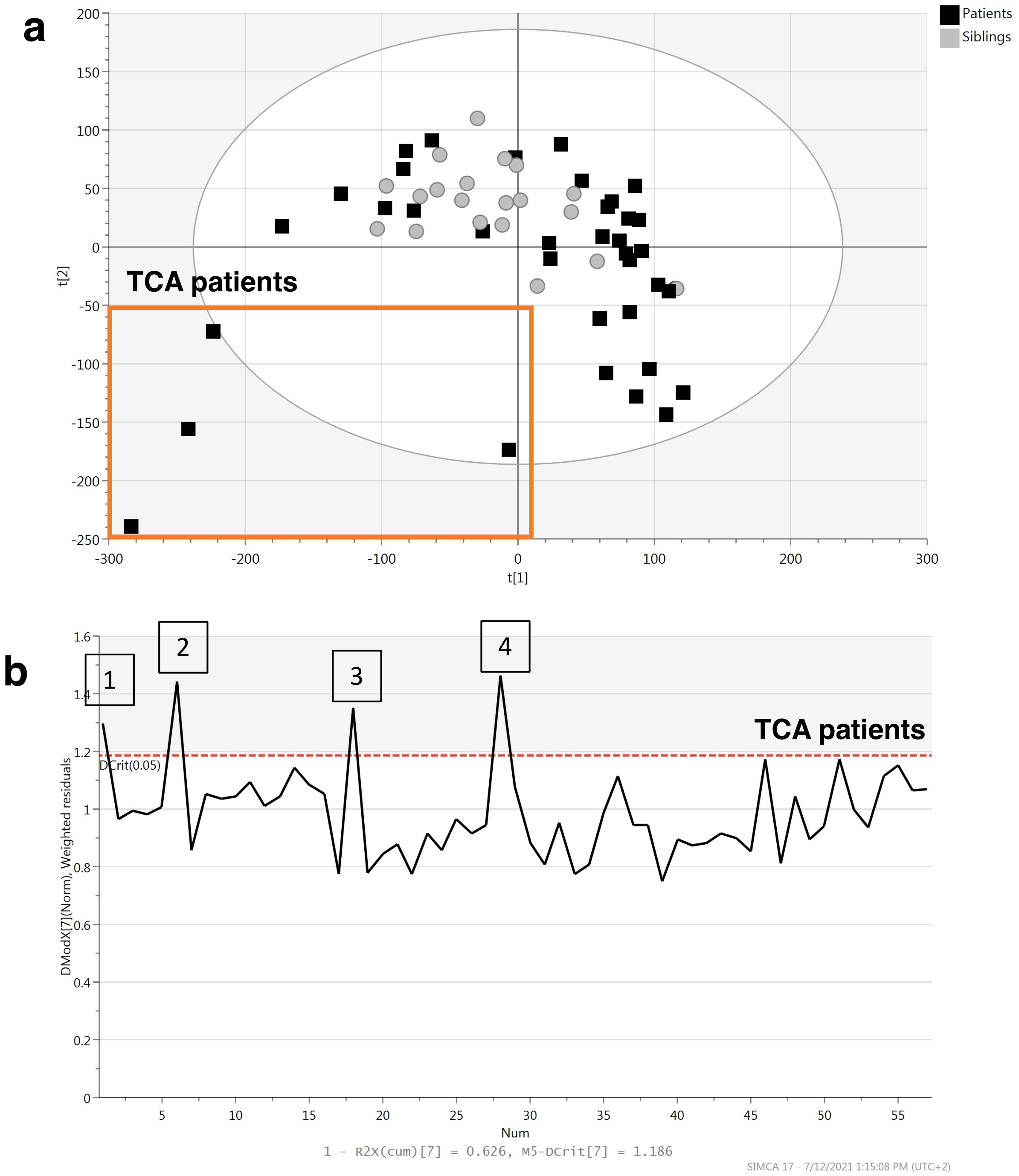

Fig 1S. Total colonic aganglionosis patients (TCA, n=4) identified as outliers in HD patient and healthy siblings cohort. a. PCA-X score-plot of patients *vs.* siblings model. TCA patients are plotted separately (shown in orange rectangle) with three out of four observations situated outside the Hotelling's T2 range (white ellipse). **b.** Distance to model plot (normalized to pooled RSD of the model) show four observations belonging to TCA patients exceeding critical limit level (Dcrit, red line), hence acting as outliers in the model.
